# Physical Demands in the Worst-Case Scenarios of Elite Futsal Referees Using a Local Positioning System

**DOI:** 10.3390/s23218662

**Published:** 2023-10-24

**Authors:** Gemma Martinez-Torremocha, Javier Sanchez-Sanchez, Antonio Alonso-Callejo, Maria Luisa Martin-Sanchez, Carlos Serrano, Leonor Gallardo, Jorge Garcia-Unanue, Jose Luis Felipe

**Affiliations:** 1 IGOID Research Group, Physical Activity and Sport Sciences Department, University of Castilla-La Mancha, 45071 Toledo, Spain; gemma.martinez@uclm.es (G.M.-T.); antonio.alonso@uclm.es (A.A.-C.); leonor.gallardo@uclm.es (L.G.); jorge.garciaunanue@uclm.es (J.G.-U.); joseluis.felipe@uclm.es (J.L.F.); 2 School of Sport Sciences, Universidad Europea de Madrid, 28670 Villaviciosa de Odón, Spain; marialuisa.martindesanpablo@universidadeuropea.es (M.L.M.-S.); carlos.serrano2@universidadeuropea.es (C.S.)

**Keywords:** team sport, competition, endurance, game analysis, physical performance

## Abstract

The aim of this study is to analyze the worst-case scenarios of professional futsal referees during the first and second half of official matches in the Spanish Futsal Cup using a Local Positioning System (LPS) for monitoring their movement patterns. Eight professional futsal referees (40 ± 3.43 years; 1.80 ± 0.03 m; 72.84 ± 4.01 kg) participated in the study. The external load (total distance, high-speed running distance and efforts, sprint distance and efforts, and accelerations and decelerations distances) of the referees was monitored and collected using an LPS. The results revealed significant differences in the worst-case scenarios of the futsal referees during the match according to the time window analyzed (*p* < 0.05). The longest time windows (120 s, 180 s, and 300 s) showed lower relative total distances in the worst-case scenarios (*p* < 0.05). The high-speed running distances were significatively higher in the first half for the 120 s (+2.65 m·min^−1^; ES: 1.25), 180 s (+1.55 m·min^−1^; ES: 1.28), and 300 s (+0.95 m·min^−1^; ES: 1.14) time windows (*p* < 0.05). No differences were found between the first and second half for the high-intensity deceleration distance (*p* > 0.05). These results will serve to prepare the referees in the best conditions for the competition and adapt the training plans to the worst-case scenarios.

## 1. Introduction

Team sports have a referee who must regulate the rules so that matches can be carried out correctly. Referees have an essential task since they must pay attention and perform precise control over the game’s every single moment [1]. For that reason, the physical demands of referees have been studied for some years thanks to Global Positioning Systems (GPS) which are easy to transport and use [2].

Throughout the years, the physical demands in football referees have been studied [1,3,4]. However, football has different characteristics than futsal [5]. Futsal is a high-intensity, intermittent team sport which is played indoors and involves short actions of high intensity with a short recovery time between efforts such as changes of direction, sprints, accelerations (Acc), and decelerations (Dec) [6]. It is a team sport with two times of 20 min each per game, at a standstill time. So, every time the ball comes out of the field, time stops until the game resumes [7]. Moreover, in futsal there are two main referees on the court, who have different functions and must have extraordinary positions on the futsal field to observe the possible infractions [5].

Football referees cover between 10 km and 12 km per game and about 15% of the total distance at a high speed (>18 km∙h^−1^) [3,4]. Furthermore, they reach a maximum speed of 28.76 km∙h^−1^ and cover 212.98 m at sprint speed [1]. Weston et al. [8] showed that the distance covered at a high intensity and the total distance travelled in the first 15 min of the first half of the match are higher than the distance covered in the first 15 min of the second half. This indicates that referees are subjected to high loads, which may cause injuries if they do not train at optimal levels that are equivalent to the physical demands that they have in official matches [9]. Nevertheless, in reference to futsal, Rebelo et al. [5] demonstrated that referees perform intermittent endurance, of moderate–high intensity, during a match, with several periods of running and sprints, while also performing long recovery periods of low intensity.

There are several studies conducted on the profiles of futsal players [7,10], but there is very little research about futsal referees [5,11,12]. It has been demonstrated that futsal players from the 1st division of the Portuguese, Spanish, and Russian leagues perform high-intensity efforts every 43 s, medium-intensity efforts every 37 s, and low-intensity efforts every 14 s during futsal playoffs’ matches [13]. In addition, a recent study published that futsal players perform around 70 Acc and Dec at a high intensity and, approximately, 170 changes of direction during official matches [14]. Also, players cover 3749 m in a match, of which 134.9 m are carried out at a high speed (>18 km∙h^−1^) [10]. Additionally, Serrano et al. [12] showed that futsal referees cover 5719 m of long distance at slow and moderate speeds, with a reduction in the second half of the games in the sprints’ distance and high-speed running (HSR). Ahmed et al. [11] demonstrated that futsal referees have 150.9 beats per minute (bpm) and cover 161.1 m and 114.4 m at a high intensity in the first and second half, respectively.

For the analysis of the physical demands of the matches of indoor and outdoor sports, different methods have been used, but GPS is the most common for providing measurements with validity and accuracy [2]. Additionally, the physical demands have been analyzed in different ways, as follows: with video for futsal referees [5,11]; via GPS for official football referees [1,3,4]; and through a Local Positioning System (LPS) with Ultra-Wide Band (UWB) technology for futsal referees [12] and players [10,15,16]. Therefore, new methods have been used to detect worst-case scenarios (WCSs). This is the case for professional football players, as using averages may underestimate peak requirements [17].

The WCS is the most intense time of a game or training [17] (e.g., 30 s). WCSs have recently started to be included in different studies [15,17] to examine the peak physical demands at different playing times during matches [18] in several team sports such as rugby [19], football [17], and futsal [15]. As a result, it would be interesting to use it for referees. However, although there is some information about the physical demands of professional futsal referees [11,12], much more evidence is needed to accurately establish their activity. Furthermore, there is no prior information on WCSs of professional futsal referees collected using tracking technology devices during official matches.

Therefore, the aim of this study is to analyze the WCSs of professional futsal referees in the first and second half of official matches in the Spanish Futsal Cup using a LPS for monitoring their movement patterns. The results of this study will facilitate the design of training plans according to the physical demands of a match.

## 2. Materials and Methods

### 2.1. Experimental Approach to the Problem

The data from seven official Spanish Futsal Cup 2020 matches (first division teams of the National Spanish Futsal League (LNFS)) were gathered using an UWB technology system. This allowed for the quantification of the absolute and relative external training loads, which were then divided into the first and second halves of the games. The competition was organized over 4 days divided into four quarterfinals’ games, two semi-finals’ games, and a final game.

### 2.2. Participants

Eight professional Spanish futsal referees (age 40 ± 3.43 years; height 1.80 ± 0.03 m; weight 72.84 ± 4.01 kg), with similar characteristics and the same number of training sessions per week, were monitored during this study. They were studied over seven games, which were spread across the quarterfinals, semifinals, and championship game over four days. The referees were selected by the National Committee of Referees (CTA) for participating in the Spanish Futsal Cup 2020. All of them had at least 6 years of experience in the first division of the National Spanish Futsal League, and they must pass different physical tests each year. Informed written consent was obtained from each participant after being informed of the study’s requirements. The project was approved and followed the guidelines established by the local institution—the Bioethics Committee for Clinical Research of the Virgen de la Salud Hospital in Toledo (Ref.: 2551;17/02/2021).

### 2.3. Equipment

The referees’ movement patterns throughout each game were tracked using WIMU PRO^TM^ LPS (RealTrack Systems SL, Almería, Spain) with an UWB technology. The reference technology and the WIMU PRO^TM^ inertial device (which the referees transported) are the two components that make up this technology. The WIMU PRO^TM^ has shown an accuracy (bias: 0.57–5.85%), test–retest reliability (%TEM: 1.19), and inter-unit reliability (bias: 0.18) in Bastida Castillo et al. [20], as well as a large ICC for the *x*-coordinate (0.65) and a very large ICC for the *y*-coordinate (0.88), with a good 2%TEM [21]. For data recording, storage, and uploading, each device has a dedicated internal microprocessor with a fast USB interface [21]. The devices are made up of a variety of sensors, including an UWB chipset with a signal frequency of 18 Hz, a gyroscope, four accelerometers, a magnetometer, and a Global Navigation Satellite System (GNSS) [22]. Six antennas make up the reference system, each of which can transmit and receive radio-frequency signals. The radio-frequency signal works almost exactly like the GPS system, with the antennas (mainly the master antenna) calculating the position of the devices in their range as they receive the calculation [21]. In terms of calculating the distance traveled, speed, mean velocity, ACC, and DEC for intermittent activities, LPS has proven to be accurate and reliable [23,24].

### 2.4. Procedures

**Figure 1:**
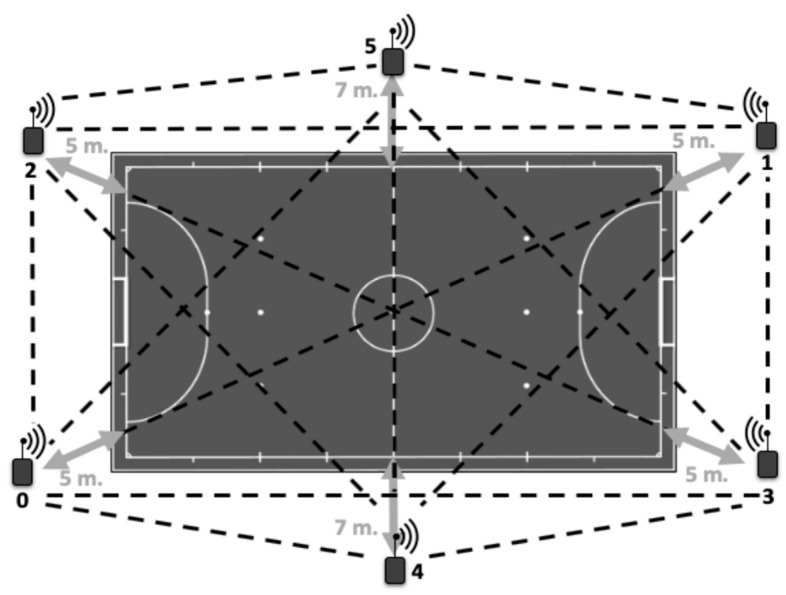
Antenna distribution of the Local Positioning System and distance reference from the futsal pitch. The arrows indicate the distance from the antennas to the court and the black lines indicate the communication between the antennas [16].

The LPS was installed on the futsal pitch where the games were played, and the individual WIMU PRO^TM^ devices (RealTrack Systems SL, Almería, Spain) were used to register the physical parameters’ data of external load. The LPS was activated after the warmup of the referees with an autocalibration of the antennas lasting 5 min [21]. The placement of the six antennas (Figure 1) was set 5 m apart, forming a hexagon, except for those positioned at the field’s middle line, which were set 7 m from the perimeter. The hexagonal shape of the antennas improved signal transmission and reception. The antennas were then self-calibrated for five minutes, while the master antenna synchronized all the antennas to a single clock after they had been installed. They were then switched on, one at a time, with the master antenna being turned on last.

### 2.5. Data Processing

The physical activity variables were considered in line with previous futsal studies [12,16]. A specific software (SPRO^TM^ v.990) was used to analyze each match’s referees’ performance data. The WCSs were assessed using the WIMU SPRO^TM^ software version 990 (RealTrack Systems SL, Almería, Spain), with a rolling average method over each physical variable selected using five different time windows (30, 60, 120, 180, and 300 s). This method had been used in previous futsal investigation [15] and other team sports’ [17,25,26]. The physical variables examined were the following: the total distance covered (TD); the HSR distance (distance covered above 15 km·h^−1^); the HSR efforts (number of efforts above 15 km·h^−1^); the sprint distance (distance covered above 18 km·h^−1^); the sprint efforts (number of efforts above 15 km·h^−1^); and the number (n) and distance (m) of high-intensity ACC (>3 m·s^−2^) and DEC (<−3 m·s^−2^). All the speed variables and Acc and Dec thresholds selected were in line with previous referee futsal research [12].

### 2.6. Statistical Analysis

The Shapiro–Wilk test was used to test for the normality of each variable and time window, resulting in a non-normal distribution (*p* < 0.05). The non-parametric Wilcoxon test for paired samples was run for each variable and time window. The same method was used for comparing values between half-times. The confidence level was set to 95%, and the *p*-values < 0.05 were considered significant. The standardized effect size was calculated for each comparison and classified as negligible (Effect Size (ES) < 0.2), small (ES between 0.2 and 0.6), moderate (ES between 0.6 and 1.2), and large (ES > 1.2). The statistical analysis was carried out and the figures were created using the RStudio software (R version 4.2.2, RStudio 2022.12.0, © 2009–2022 Posit Software, PBC).

## 3. Results

**Figure 2:**
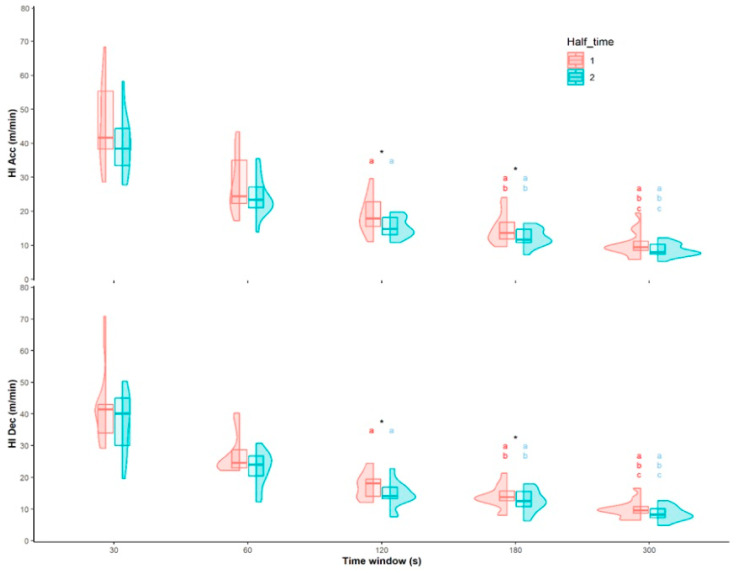
Worst-case scenarios of the elite futsal referees in the different time windows in accelerations and decelerations. * Significant differences between halves. ^a,b,c^ Significant differences between time windows [30 (a), 60 (b), 120 (c), 180 (d), 300 (e)].

The results revealed significant differences in the WCS of futsal referees during the match according to the time window analyzed (*p* < 0.05). The longest time windows (120 s, 180 s, and 300 s) showed lower relative distances in the WCS in comparison to the shortest intervals (Table 1; *p* < 0.05). According to the differences between the first and the second half in the WCS (Table 2), the HSR distance was significatively higher in the first half for the 120 s (+2.65 m·min^−1^; ES: 0.38), 180 s (+1.55 m·min^−1^; ES: 0.29), and 300 s (+0.95 m·min^−1^; ES: 0.27) time windows (*p* < 0.05). The WCS for the total distance was only significatively lower in the second half for the 120 s time window (−7.29 m·min^−1^; ES: 1.44). Finally, the high intensity Acc distance was also higher in the first half, but only for the 120 s (+3.71 m·min^−1^; ES: 0.87) and 180 s (+2.68 m·min^−1^; ES: 0.72) time windows (*p* < 0.05; Figure 2). No differences were found between the first and second half for the high intensity Dec distance (*p* > 0.05).

## 4. Discussion

The findings of this study revealed interesting results which deserve further investigation. The aim of this research was to analyze the WCS in professional futsal referees in the first and second half of the match in the Spanish Futsal Cup using an LPS. To our knowledge, this is the first study that examined the WCS during official matches in the Spanish Futsal Cup and futsal in general. The main findings of the study were that there is an extreme influence on the time window analyzed, which confirmed that the longer the time analyzed, the lower the physical demands’ requirement is during the match. Additionally, the referees covered longer high-intensity distances and more high-intensity Acc in the first half of the matches.

Previous studies have analyzed the physical demands of futsal referees [5,11,12,27] and futsal players [15,16]. The results of this research determined the physical demands without the time windows, except for the study of Illa et al. [15], which researched the positional differences in the WCS of elite futsal players. Therefore, there are studies on the physical demands of referees with similar results, although they do not analyze the WCS.

Our study highlights some interesting results. Curiously, all the variables obtained a decreasing result among the different time windows in the first and the second half of the match. These results have important practical applications for training design, as referees might have shown fatigue during the match. Nevertheless, this fatigue may have been due to the contextual variables of the match, which should also be studied in the future to determine whether they really interfere with the results.

In comparison to previous research, similarities were found in the total distance, as it decreases with time over the course of the match. This study, in contrast with Illa et al.’s [15], showed that, during the WCS of the match play, the futsal players cover more distance than the futsal referees for each time window (30 s: 37%, 60 s: 34%, 120 s and 180 s: 31%, and 300 s: 43%). An explanation for these differences might be the continuous displacement of the players as they are moving constantly or even the type and categories of the match, although, in this case, it was the same division in the same country. Another possible reason for the differences is substitutions: while a player can rest during the match, the referee cannot; however, they do not start under the same circumstances. On the other hand, we agree with Illa et al. [15] that the WCS decreases as the time window analyzed increases; this may be due to fatigue occurring during the match. Moreover, Ahmed et al. [11], in the comparison of performance between halves of a match, determined a decline in the total distance cover by the Iraq Futsal Premier League referees (3093 m vs. 2850 m), while the findings in this study reported a similar decrease in the relative total distances covered in both halves in the different time windows. The total distance is also analyzed by Serrano et al. [12] (2888.39 ± 122.55 vs. 2831.51 ± 150.26 m), showing similarities to this study in the decrement of the variable and, also, with Ahmed et al. [11]. The physical demands were monitored using different devices in Ahmed et al. [11], so this could explain the little difference with the total results. Moreover, the competitions were different and, therefore, the contextual demands were also dissimilar. In addition, there are studies that look at the relative distances of elite futsal players in official matches, showing that they have similar values between halves [16] or even experience an increase [10] compared to the results of this research. The contextual variables during the matches might be the reason for these differences, as well as the kind of competition, since the referees must cover the different distances depending on the course of the match.

Furthermore, futsal referees cover more distance at sprint speed (>18 km∙h^−1^) during the WCS of the match play (30 s: +8%, +16%; 60 s: +5%, 15%; 120 s: +6%, +12%; 180 s: +6%, +22%; and 300 s: +5%, +17%) than futsal defenders and pivots during the match in all time windows, but cover less distance than wingers (30 s: −5%, 60 s: −13%, 120 s: −17%, 180 s: −17%, and 300 s: −31%) [15]. However, Illa et al. [15] only described the HSR as >18 km⋅h^−1^, while in this study it is studied as >15 km⋅h^−1^ and the sprint as >18 km⋅h^−1^. The differences in the distance covered at a high intensity by the referees and players may be due to the specific nature of their individual roles during the matches. The referees must always follow the game, as being too far away from fouls may result in incorrect decisions. Even so, rugby union players [19] had lower values in the longest time window than the futsal referees (−6%). The reason for the dissimilarity with rugby union players might be due to the interruptions during the futsal matches (every action that stops the match clock), in which the futsal referees and players continue moving even when the match is detained, for example when there are outsides, corner-kicks, etc. [28]. Nevertheless, Serrano et al. [12] did not study the WCS but showed that referees covered less distance at HSR (>15 km⋅h^−1^) in the second half (235.06 ± 67.58 m vs. 207.78 ± 57.86 m, respectively). The same occurred in this study, as the distance travelled at HSR decreased from the first to the second half based on the time windows. The specific situations of futsal matches may slow down the action in the second half, which could have an impact on the referees’ ability to handle these demands. Moreover, the number of actions over 18 km∙h^−1^ during matches is higher in the referees, in the lower windows (30 s, 60 s, and 120 s), than in the players in the different positions; however, in the longer windows (180 s and 300 s), the wingers have a higher number of such actions [15]. Nevertheless, the course of the match and the type of competition of futsal matches might also explain the differences in the results. Another possible explanation for this difference could be that they have a wider range of motion to achieve a greater speed. Additionally, the sprint distance of the present results showed a lower sprint distance (>18 km∙h^−1^) in the second half from every time window, as occurred in Serrano et al.’s study [12], although they did not study the WCS.

On the other hand, the Acc and Dec variables were barely discussed in this study. However, these results can be discussed using previous studies. Acc and Dec are an important part of the physical demands of futsal referees since the number of high-intensities Acc and Dec is very high during the matches [12]. Other studies have analyzed stops, sideways running, and turns during matches [5,11] to report these important actions, as they are one of the causes of sport injuries. While previous studies have examined the results as whole, without taking the WCS into account [5,11,12], the result of the present study shows values of high-intensity Acc and Dec distances of the different time windows. In addition, the high-intensity Acc and Dec values show a decrease in the second half; these results are similar to the results of a previous futsal referees’ study [12]. Therefore, high-intensity Acc and Dec require a high eccentric force which may produce muscular fatigue, so monitoring them could be useful for designing strength training and injury-prevention programs.

Player performance was not examined in the current study, but player activity and game development may affect compliance with these requirements because referee activity is influenced by the match activity [11]. Regardless, based on the data obtained in this study, training programs should be adjusted to the characteristics of the specific competition to improve referees’ physical performance and prevent injuries. Illa et al. [15] had studied the seasonal trend on all the dependent variables for the different positions, which may be useful for developing training plans to prevent injuries. So, this could be a future, possible focus for research on futsal referees.

This research had different limitations, as there was a low number of matches and referees involved. Nevertheless, the study contains all the Spanish Futsal Cup matches.

Understanding the physical profile and performance of the referees may be aided by the potential correlation between the physical requirements placed on the players during competitions and the referees’ physical parameters [11,29].

## 5. Conclusions

Finally, the use of LPS to monitor physical performances provides knowledge of the specific activity profiles of futsal referees. This information could be useful for making more accurate training programs and even for developing new physical tests. With all of this, referees will know their workload requirements for each match during the different seasons and, also, it will serve them to be able to have different references so that they can be in the best physical conditions to face the different matches.

One of the most significant findings to emerge from this study is the extreme effect of the time window, which confirmed that the longer the time analyzed, the lower the requirement is during the matches. Therefore, time windows can be used in different ways, depending on their length. Shorter time windows (30 s, 60 s and 120 s) are useful for designing high-intensity tasks in short periods of time, while longer time windows (180 s and 300 s) are effective for tasks that are performed over larger spaces where extensive, high-intensity actions are to be performed.

In addition, the present study also identified that referees have their best results in the first half of the matches over the longer time windows. The decline in their physical performance in the second half may be attributed to the referees’ need to keep up with the players’ pace and the situations that arise during the futsal matches. This could lead to a reduction in the intensity of the match in the second half, which would affect the performance of the referees.

Finally, it should be considered that it could be helpful to study contextual variables when carrying out future studies. Moreover, these results will serve to prepare referees in the best conditions for the competition and allow to adapt the training plans to critical match scenarios that may be accompanied by relevant decision making.

## Figures and Tables

**Table 1:** Worst-case scenarios of the elite futsal referees in the different time windows.

Time Window (s)	30 (a)		60 (b)		120 (c)		180 (d)		300 (e)	
n = 28	Mean	SD	Mean	SD	Mean	SD	Mean	SD	Mean	SD
Total distance (m·min^−1^)	143.30	14.61	115.39 ^a^	9.28	94.47 ^a,b^	6.21	87.20 ^a,b,c^	4.76	66.22 ^a,b,c,d^	3.22
HSR Distance (m·min^−1^)	66.44	17.73	40.23 ^a^	11.33	24.21 ^a,b^	7.06	18.90 ^a,b,c^	5.38	12.89 ^a,b,c,d^	3.40
HSR count (n·min^−1^)	6.79	2.27	4.00 ^a^	1.12	2.64 ^a,b^	0.61	2.13 ^a,b,c^	0.55	1.48 ^a,b,c,d^	0.27
Sprint Distance (m·min^−1^)	51.21	15.81	28.38 ^a^	10.90	17.11 ^a,b^	6.67	12.78 ^a,b,c^	4.80	8.39 ^a,b,c,d^	3.06
Sprint count (n·min^−1^)	5.57	2.13	3.14 ^a^	1.35	1.95 ^a,b^	0.70	1.48 ^a,b,c^	0.57	1.00 ^a,b,c,d^	0.36
HI Acc Distance (m·min^−1^)	42.46	10.67	26.20 ^a^	7.34	17.21 ^a,b^	4.60	13.63 ^a,b,c^	3.90	9.48 ^a,b,c,d^	3.03
HI Dec Distance (m·min^−1^)	39.79	10.83	24.99 ^a^	6.30	16.01 ^a,b^	4.20	13.34 ^a,b,c^	3.53	9.31 ^a,b,c,d^	2.56

^a,b,c,d^ Significant differences between time windows [30 (a), 60 (b), 120 (c), 180 (d), 300 (e)].

**Table 2:** Worst-case scenarios of the elite futsal referees in the different time windows by half-time.

	Time Window (s)	30 (a)		60 (b)		120 (c)		180 (d)		300 (e)	
	n = 14	Mean	SD	Mean	SD	Mean	SD	Mean	SD	Mean	SD
First Half	Total distance (m·min^−1^)	144.90	12.72	116.42	8.91	98.12 *^,a^	5.15	88.32 ^a,b^	4.99	66.43 ^a,b,c^	2.76
	HSR Distance (m·min^−1^)	66.15	15.64	41.35	9.72	25.54 *^,a^	5.87	19.68 ^a,b^	4.92	13.36 *^,a,b,c^	2.66
	Sprint Distance (m·min^−1^)	51.93	15.01	29.08	10.27	17.59 ^a^	6.10	13.38 ^a,b^	4.45	8.71 ^a,b,c^	2.63
	HI Acc Distance (m·min^−1^)	45.44	11.99	28.24	8.49	19.06 *^,a^	5.30	14.97 *^,a,b^	4.59	10.39 ^a,b,c^	3.72
	HI Dec Distance (m·min^−1^)	42.32	11.88	27.43	6.33	17.38 ^a^	4.07	14.03 ^a,b^	3.62	10.06 ^a,b,c^	2.72
Second Half	Total distance (m·min^−1^)	141.71	16.61	114.35	9.85	90.83 ^a^	4.98	86.07 ^a,b^	4.41	66.01 ^a,b,c^	3.72
	HSR Distance (m·min^−1^)	66.73	20.21	39.12	13.03	22.89 ^a^	8.08	18.13 ^a,b^	5.89	12.42 ^a,b,c^	4.07
	Sprint Distance (m·min^−1^)	50.49	17.11	27.68	11.85	16.62 ^a^	7.40	12.18 ^a,b^	5.21	8.07 ^a,b,c^	3.51
	HI Acc Distance (m·min^−1^)	39.48	8.59	24.17	5.57	15.35 ^a^	2.90	12.29 ^a,b^	2.58	8.58 ^a,b,c^	1.88
	HI Dec Distance (m·min^−1^)	37.25	9.40	22.55	5.44	14.62 ^a^	4.00	12.64 ^a,b^	3.43	8.56 ^a,b,c^	2.23

* Significant differences between halves. ^a,b,c^ Significant differences between time windows [30 (a), 60 (b), 120 (c), 180 (d), 300 (e)].

## Data Availability

Due to the privacy terms, the data are not available.
